# The cost of diversity in livestock feed rations

**DOI:** 10.1371/journal.pone.0277817

**Published:** 2022-11-17

**Authors:** Adam M. Komarek, Sherman Robinson, Daniel Mason-D’Croz

**Affiliations:** 1 School of Agriculture and Food Sciences, The University of Queensland, Gatton, QLD, Australia; 2 International Food Policy Research Institute, Washington, DC, United States of America; 3 Department of Global Development, College of Agriculture and Life Sciences, Cornell University, Ithaca, NY, United States of America; International Maize and Wheat Improvement Centre: Centro Internacional de Mejoramiento de Maiz y Trigo, MEXICO

## Abstract

This study investigates the financial cost of increasing the diversity of cereal grains in livestock feed rations. We first develop a nonlinear mathematical programming model that determines the least-cost composition of livestock feed rations of one metric ton that have at least the same energy and nutrient content as a reference feed ration. We then add into the model a diversity constraint using the Simpson Index of diversity to examine how changes in the diversity of the commodities in the ration affect the cost of the ration while maintaining the ration’s energy and nutrient content at a reference ration value. We apply the model to cereal grain feed rations for livestock in 153 countries, using reference rations that depict the historical composition of cereal grain feed rations offered to livestock in each country. Results suggest that a one percent change in ration diversity changed the ration cost (*i*.*e*., the cost-diversity elasticity) from −0.67% to 1.41% (average = −0.02%) across all countries. Our results suggest that changes in ration diversity can come at a financial cost, but this financial cost appears negligible in many countries. This negligible cost could provide the feed sector more encouragement to diversify its feed supply and potentially become more resilient to price and production shocks.

## 1. Introduction

The original formulation of the diet problem identifys a least-cost combination of foods that meet the nutritional needs of a person [1]. The standard approach in mathematical programming models of the diet problem is that the least-cost solution reveals a level of diversity by setting minimum intakes for nutrients as constraints. Inevitably the solution requires several foods to meet the constraints at least cost. In this study, we develop a nonlinear mathematical programming model that starts from the diet problem to describe a problem of developing a least-cost livestock feed ration and then incorporate diversity into this model. Our model examines the explicit cost of diversity that is often implicit in least-cost solutions. We applied our model to ask the question how changes in diversity in livestock feed rations affect the costs of livestock feed rations for a decision maker who supplies, at least cost, a minimum level of energy and nutrients? The model is an optimization model that minimizes feed ration costs for a one metric ton ration subject to constraints on minimum levels of energy and nutrients in the feed ration.

Although the economic benefits of specialization, such as lowering average costs, are well established [2], there may also be benefits from firm diversification related to risk management. As an example, if supply is more uncertain then demand, multiple sourcing is a common risk management strategy in supply chains [3]. Despite researchers having studied the benefits of diversity in agricultural production systems (examples provided in Section 2), the cost of reaching a level of diversity remains less studied. This lack of emphasis on the cost of diversity is a concern; the laws of economics also apply to diversity and greater diversity in agricultural production systems may have opportunity costs and tradeoffs elsewhere [4], either for society or individuals.

Our approach of introducing a measure of diversity as a constraint in a least-cost model provides a framework for quantifying the cost of supplying a livestock feed ration, with a minimum content of energy and nutrients, at different levels of feed commodity diversity. Our study embeds into a nonlinear programming model an approach to calculate the cost of increasing diversity using the Simpson Index. The cost of diversity is important to quantify because although diversity is of value in many contexts, it may come at a cost, and we calculate this cost using an approach that allows for consistent cost comparisons between countries. Rather than restrict the mass of the individual commodities in the one metric ton feed ration to within a specific range, we use a diversity index that places constraints on the level of diversity in the feed ration, thereby giving a nonlinear problem.

Section 2 describes a livestock feed ration problem and discusses how preferences for diversity may differ between producers and consumers. We then present our nonlinear programming model in Section 3.1. In the nonlinear programming model, we parametrically add a diversity constraint using the Simpson Index that solves a problem similar to the diet problem for humans. We apply the model in Section 3.2 for 153 countries in which livestock were offered cereal grain (hereafter grain), based on a global dataset [5].

## 2. Livestock feed ration problem

Feed prices can change because several factors, including weather variability, changes in food demand, and changes in environmental and trade policies [6–9]. These feed price changes may have a profound influence on the composition of feed rations and the profitability of livestock-related activities. Over time livestock diets have shifted towards including more grains [10], and this trend is expected to continue [11]. Decision makers in the livestock sector, such as large commercial feed manufacturers and individual livestock producers, often attempt to minimize the cost of producing a predetermined output [10, 12]. An example would be a large commercial feed manufacturer choosing a set of commodities that minimizes the cost of producing a bag of cereal grain feed concentrate of a specified energy and nutrient content. This bag would be an intermediate product in the production of livestock products such as meat, milk, eggs, and wool. Mathematically, this choice problem is similar to the diet problem for humans.

An enduring literature has applied mathematical programming models to identify least-cost diets consistent with dietary standards for both humans and livestock. The diet problem has mostly been solved as a linear programming model, with the simplest model minimizing cost subject to nutrient constraints. The simplest model often generates solution diets that differ from observed diets unless the model is constrained in specific ways. Studies examining the diet problem abound for both humans [13–20] and livestock [21–24]. From the diet problem literature, realistic models often deviate from the diets generated in programming models to give solutions with higher costs and more diverse diets for both humans and livestock. Humans value dietary diversity for a variety of reasons, which include, within specific ranges, food diversity contributing to nutritional adequacy [25] and food security [26], especially in developing countries. Production diversity in agricultural production systems can improve food availability by providing functional redundancy [27, 28], help manage on-farm risks [29, 30], and provide greater stability in the flow of products through supply chains [31, 32]. Despite these benefits, less studies have examined the costs associated with increased diversity in livestock feed rations.

One approach to bringing diversity into studying livestock feed rations is to use indexes of diversity as constraints in a programming model. For humans, a desire for diversity is based on the utility function of consumers. Dixit and Stiglitz [33] writes “the convexity of indifference surfaces of a conventional utility function defined over the quantities of all potential commodities already embodies the desirability of variety.” (p.297). For livestock, producers of feed rations may care more about feed diversity within their enterprises than whether livestock prefer consuming a more diverse feed ration. Appropriate measures of desirable diversity will differ for the two cases. For example, a measure of dietary diversity for humans is to count the number of foods or food groups consumed over a recall period [26]. Price indices have also been developed to track the cost of achieving a minimum level of dietary diversity over time for humans, based on consuming at least five different food groups [34]. If a person consumes any amount of each food or food group from the available options, then the person achieves maximum diversity. However, this approach does not provide any information about the distribution of individual foods consumed. Several indexes based on continuous variables have been applied to measure diversity such as the Simpson Index [35], Shannon index [36], and the Herfindahl-Hirschman Index [37, 38].

## 3. Methods

### 3.1. Model

Consider a producer who supplies a feed ration with a specific nutrient content to a consumer. Nutrient content is defined as the nutrients per unit of mass. This producer could be a manufacturer of a livestock feed ration like Cargill who purchases commodities as inputs into their feed mill production process on current or futures markets. The consumer could be a feedlot manager who buys the feed ration to then feed to livestock. Individual feeds in the feed ration can be classified into four types: 1) grain, usually fed as concentrates, 2) grass and silage, 3) occasional feed, and 4) stover (fibrous crop residues) [5]. A feed ration can include multiple individual feeds from one or more of the four feed types, for example the model could minimize the cost of a one metric ton grain feed ration, depending on the data available and the context of the producer. Hereinafter a feed is referred to as a commodity (*c*).

The producer has a choice of commodities to combine to produce a feed ration. For example, a one metric ton grain feed ration could be composed of maize, sorghum, and wheat, and each commodity may have a different mass contributing to the one metric ton. The consumer has information on the nutrient content of the product, such as on a label for a bag of cereal grain feed concentrate, or through a contract specifying the nutrient content. The producer aims to minimize the financial cost of obtaining the one metric ton feed ration by choosing the mass of each commodity (*x_c_*) subject to the price and nutritive value of each individual commodity. The ration cost can be written as

$$\sum_{c=1}^{C}p_{c}x_{c}$$ (1)

where in Eq (1) *p*_*c*,_ is the price of commodity *c* ($ kg^−1^), *x_c_* is the mass of the commodity in kg, and *C* is the total number of commodities available. The mass of each commodity is non-negative (Eq 2):

$$x_{c}\geq 0,c=1,\ldots ,C.$$ (2)

The total mass of all the individual commodities in the cereal grain feed ration is no more than one metric ton (Eq 3):

$$\sum_{c=1}^{C}x_{c}\leq 1000.$$ (3)

The producer aims to minimize the ration cost subject to the nutrient content of the feed ration being at least equal a target value. The target value in our study is derived from a reference ration, which identified the mass of individual grains typically offered to livestock in a country (Section 3.2). Eq (4) specifies the nutrient content constraint for the feed ration,

$$\sum_{c=1}^{C}a_{nt,c}x_{c}\geq b_{nt},nt=1,\ldots ,N,$$ (4)

where *a_nt,c_* is the nutritive value of the *N^th^* nutrient *nt for commodity c*. The right-hand side of Eq (4), *b_nt_,* is the nutrient content of the reference ration for the *N^th^* nutrient *nt*. The numerical value of *b_nt_* is set by summing across each commodity in the reference ration the mass of each commodity and that commodity’s nutritive value per unit of mass. Ration costs only relate to the purchasing of commodities to achieve the required nutrient content of the feed ration. This focus on commodity purchasing costs implies that other production-related costs such as maintenance of equipment and electricity remain unchanged if the feed ration changes. We used commodity shares that can range from zero to one as the first step to calculate diversity. Eq (5) calculates the share (*s_c_*) of each commodity in the feed ration,

$$s_{c}=\frac{x_{c}}{\sum_{c=1}^{C}x_{c}},$$ (5)

where *s*_*c*_ is the share of the c*^th^* commodity in the feed ration and the shares sum to one (Eq 6),

$$\sum_{c=1}^{C}s_{c}=1.$$ (6)

We calculated the diversity of the feed ration using the Simpson Index (Eq 7). The Simpson Index incorporates richness and evenness and is amendable to zero quantities. Richness refers to the number of commodities in the feed ration and evenness refers to how uniformly the individual commodities are distributed within the feed ration.


$$Simpson\;Index\;(SI)\;of\;diversity=1-\sum_{c=1}^{C}s_{c}^{2}$$ (7)

The Simpson Index has a theoretical range of zero to one. A Simpson Index of zero indicates the feed ration includes only one commodity, which is the least diverse ration possible. The upper limit of the Simpson Index is $1-\frac{1}{C}$, and as *C* approaches infinity the Simpson’s Index approaches one, which is the most diverse ration possible.

To study the cost of diversity we first identified combinations of commodities that generated the minimum and maximum Simpson Index feasible in each country that also maintained the nutrient content of the ration in each country. To calculate the ration cost for varying levels of diversity we constrained the Simpson Index in equal numerical steps (*n* = 0, 1, 2,..,*N*) with each step increasing the Simpson Index by $\frac{n}{N}$. We constrained the Simpson Index to values between its minimum (*SI_min_*) and maximum (*SI_max_*) feasible value using Eq (8), given the other model constraints.


$${SI}_{n}=\sum_{n=0}^{N}({SI}_{min}+(({SI}_{max}-{SI}_{min})\times \frac{n}{N})),n=0,1,\ldots ,N.$$ (8)

Where in Eq (8), *SI_n_* is the Simpson Index at step *n*. Eq (8) is used to constrain the Simpson Index to a specific value. Solving the model for all *N* captures the range of feasible levels of diversity. Our model solves explicitly for the mass of each commodity that provides a least-cost ration with a minimum energy and nutrient content as the level of commodity diversity in the ration varies.

### 3.2. Application

Our study applied versions of the above nonlinear programming model, Eqs (1) to (8), to cereal grain feed rations (hereinafter feed ration or ration) and the model is solved at the country-scale on an annual time step across the globe using the series of scenarios described in Table 1. The model selected the mass of each *x_c_* from a possible seven cereal grains by first solving nonlinear programming models to identify *SI_min_* and *SI_max_*. The model then selected the mass of *x_c_* by solving least cost feed rations for diversity between *SI_min_* and *SI_max_*. The nonlinear programming model is written in the General Algebraic Modeling System version 36.2.0. The model is solved using the generalized reduce gradient algorithm CONOPT solver [39] as the first solver option and the IPOPTH solver as the second solver option.

**Table 1:** Summary of scenarios simulated in each country using the mathematical programming model.

Item	Scenario			
	Minimize diversity without cost considerations	Maximize diversity without cost considerations	Minimize cost without diversity constraint	Minimize cost with diversity constraint
Nutrient content of feed ration	At least equal to reference ration for all nutrients and energy	At least equal to reference ration for all nutrients and energy	At least equal to reference ration for all nutrients and energy	At least equal to reference ration for all nutrients and energy
Restriction on Simpson Index of diversity	Between 0 and $\frac{6}{7}$	Between 0 and $\frac{6}{7}$	Between 0 and $\frac{6}{7}$	Set between the minimum feasible diversity and maximum feasible diversity using 10 equal incremental steps
Commodity costs	No costs considered	No costs considered	Costs are part of objective function	Costs are part of objective function
Scenario objective	Minimize diversity	Maximize diversity	Minimize cost	Minimize cost
Model equations	Minimize the value of Eq (7) subject to Eqs (2)–(6)	Maximize the value of Eq (7) subject to Eqs (2)–(6)	Minimize the value of Eq (1) subject to Eqs (2) to (6)	Minimize the value of Eq (1) subject to Eqs (2)–(8) with N = 10
Model choice	Select mass of each commodity	Select mass of each commodity	Select mass of each commodity	Select mass of each commodity

*Notes*: Reference ration data are country-specific commodity mixes based on the share of each cereal grain commodity in cereal grain feed rations [5]. In all scenarios the feed ration mass is no more than one metric ton.

We used the model solutions to calculate the cost-diversity elasticity using the values of ration costs and ration diversity one *n* step above and one *n* step below the reference ration cost-diversity combination. This cost-diversity elasticity is the percentage change in ration cost in response to a given percentage change in the Simpson Index of diversity. The cost effect of an increase in feed ration diversity is an empirical question, but *ex-ante* several factors may influence the sign of the cost effect including if there are any per unit input cost advantages realized as diversity increases. A typical long run average cost curve is U-shaped where average costs initially decline as output increases and eventually reach a turning point after which additional output results in average costs increasing. Similarly, it might be the case that the relationship between cost and diversity is also U-shaped where at the extremes of low and high diversity the costs are higher than at some intermediate level, but this would be an empirical question.

Data for our application came from three sources. First, a global feed dataset that reported the mass of individual grain commodities offered to all cattle, small ruminants, pigs, and poultry in the year 2000 at the country scale for 153 countries [5]. Second, data on the energy content and nutritive content of each grain price [40]. Third, data on the price of each grain at the country scale [41].

The global feed dataset provided the total mass of each grain fed to livestock at the country scale per year. We used these data to compute standardized one metric ton livestock feed rations at the country scale. We then computed the share of each grain in the standardized one metric ton livestock feed rations. This share is equivalent to the percentage contribution by mass of an individual grain to the total mass of the one metric ton ration. Taken together the shares and total mass of each grain in the one metric ton feed rations [5] are considered our reference ration. The seven grains considered were barley, maize, millet, sorghum, rice, wheat, and other. The dataset we used included some unknown grains. The nutrient content of these unknown grains was unknown, we therefore assigned the unknown grains the average nutrient content of major grains not included in the reference ration. In our study, these unknown grains represented a composite of grains (labelled ‘other’ in our results) that included oats and rye, along with pseudocereals such as buckwheat. We used the average price of oats and rye from OECD [42] as a proxy price for the unknown grain. The nutrient content of the unknown grain was the weighted average of oats and rye from Feedipedia [40]. The weighting used in the weighted average was related to the global mass of oats and rye offered to livestock in the year 2000 [43].

Table 2 reports the energy and nutritive value of each grain used in our study, which was the same in each country. Our programming model considered energy and 10 nutrients in the *nt* in Eq (4): (crude) protein, calcium, copper, iron, magnesium, manganese, phosphorus, potassium, sodium, and zinc.

**Table 2:** Energy and nutritive value of the grains used in the study.

Item	Units	Crop							
		Barley	Maize	Millet		Rice	Sorghum	Wheat	Other
Gross energy	MJ	18.4	18.7		17.7	17.6	18.8	18.2	19.0
Crude protein	g	118	94		89	83	108	126	108
Calcium	g	0.8	0.5		4.9	0.6	0.3	0.7	1.0
Copper	mg	12.0	2.0		7.0	3.0	5.0	6.0	3.0
Iron	mg	184.0	37.0		1208.0	53.0	120.0	78.0	110.0
Magnesium	g	1.3	1.2		1.8	1.0	1.8	1.2	1.0
Manganese	mg	19.0	5.0		0.0	82.0	12.0	40.0	34.6
Phosphorus	g	3.9	3.0		3.4	2.9	3.3	3.6	3.6
Potassium	g	5.7	3.9		5.3	2.8	4.3	4.6	4.9
Sodium	g	0.1	0.0		0.0	0.3	0.2	0.0	0.1
Zinc	mg	30.0	21.0		31.0	14.0	24.0	31.0	25.7

*Notes*: The values in the “Units” column are read as the stated unit per kg of dry matter. MJ is megajoules, g is grams, and mg is milligrams. Values for each crop refer to the grain component of the crop. Data from Feedipedia [40].

We reported feed ration costs and diversity levels for the feed rations by country income group, using the World Bank [44] Country Group classification. This classification divides countries into groups based on Gross National Income per capita for the financial year 2018. The four groups were: low income, lower-middle income, upper-middle income, and high income. We reported our simulation results for ration costs, diversity levels, and commodity mixes by region using the seven regions from World Bank [44]: East Asia and Pacific, Europe and Central Asia, Latin America & the Caribbean, Middle East and North Africa, North America, South Asia, and Sub-Saharan Africa. For reporting simulation results, we selected the two countries in each of the seven regions with the highest gross financial value of livestock production in the year 2000 [43]. The fourteen countries were: Australia and China in East Asia and Pacific, Germany and the Russian Federation (hereinafter Russia) in Europe and Central Asia, Argentina and Brazil in Latin America & the Caribbean, the Arab Republic of Egypt (hereinafter Egypt) and the Islamic Republic of Iran (hereinafter Iran) in Middle East and North Africa, Canada and the United States of America (hereafter United States) in North America, India and the Islamic Republic of Pakistan (hereinafter Pakistan) in South Asia, and Sudan and South Africa in Sub-Saharan Africa. The fourteen countries selected are used for illustrative purposes to report graphical results in more details than could be graphically reported for all 153 countries.

We used country-specific prices in our model for constant 2005 $ (purchasing power parity adjusted). These prices include any country-specific tariffs, producer support estimates, transport costs and marketing costs [45]. The prices used were those derived from simulation analysis that used the International Model for Policy Analysis of Agricultural Commodities and Trade [41]. Table 3 reports the prices in the fourteen selected countries and summary, non-inferential, statistics for all countries. We studied cereal grains because their market prices are available, and our study application is most applicable to commercial cereal grain feed markets, rather than smallholder farmers who may be a producer and consumer of a cereal grain. In contrast, other types of livestock feed, such as grasses or stover, are rarely traded in formal markets and therefore market prices for them rarely exist.

**Table 3:** Grain prices used in fourteen select countries and summary statistics of prices used in all 153 countries.

				Barley	Maize	Millet	Rice	Sorghum	Wheat	Other
Region	Country	Income group	Statistic	(constant year 2005 $ metric ton^-1^)						
East Asia & Pacific	Australia	High income	Actual	199.32	170.86	319.14	536.77	161.24	191.45	165.25
East Asia & Pacific	China	Upper middle income	Actual	215.93	185.10	345.74	637.41	174.67	207.41	179.02
Europe & Central Asia	Germany	High income	Actual	199.32	170.86	319.14	536.77	161.24	191.45	165.25
Europe & Central Asia	Russia	Upper middle income	Actual	232.54	199.34	372.33	738.06	188.11	223.36	192.79
Latin America & Caribbean	Argentina	Upper middle income	Actual	215.93	185.10	345.74	637.41	174.67	207.41	179.02
Latin America & Caribbean	Brazil	Upper middle income	Actual	215.93	185.10	345.74	637.41	174.67	207.41	179.02
Middle East & North Africa	Egypt	Lower middle income	Actual	232.54	199.34	372.33	738.06	188.11	223.36	192.79
Middle East & North Africa	Iran	Upper middle income	Actual	232.54	199.34	372.33	738.06	188.11	223.36	192.79
North America	Canada	High income	Actual	199.32	170.86	319.14	536.77	161.24	191.45	165.25
North America	United States	High income	Actual	199.32	170.86	319.14	536.77	161.24	191.45	165.25
South Asia	India	Lower middle income	Actual	215.93	185.1	345.74	637.41	174.67	207.41	179.02
South Asia	Pakistan	Lower middle income	Actual	215.93	185.1	345.74	637.41	174.67	207.41	179.02
Sub-Saharan Africa	South Africa	Upper middle income	Actual	199.32	170.86	319.14	536.77	161.24	191.45	165.25
Sub-Saharan Africa	Sudan	Lower middle income	Actual	249.15	213.58	398.93	838.7	201.55	239.31	206.56
All	All	All	Average	225.13	192.99	349.20	693.08	176.34	216.24	186.65
All	All	All	Minimum	167.75	143.81	268.61	338.83	135.71	161.13	139.08
All	All	All	Maximum	249.15	213.58	398.93	838.70	201.55	239.31	206.56

*Notes*: Data sourced from a global simulation study [41]. All prices are inclusive of country-specific tariffs, producer support estimates, and marketing margins [45].

## 4. Results

Table 4 reports the reference ration number of grains, costs, and diversity level. Costs were higher in low-income countries and were also more variable. The cost per metric ton across the high-income countries ranged from $171 to $435 (average = $198), compared to a range in low-income countries for the cost per metric ton from $176 to $781 (average = $284). Ration cost variability, calculated as the coefficient of variation (defined as the standard deviation divided by the average), was 0.21 in the high-income countries and was 0.49 in the low-income countries. The diversity of the feed ration, calculated using the Simpson Index, was higher in higher income countries (Table 4) than in low-, low middle-, and upper middle-income countries. The average Simpson Index was 0.56 in high-income countries and 0.29 in low-income countries. The number of grains in each feed ration, in general, increased as incomes increased, and averaged 3.04 in low-income countries, 4.21 in lower middle-income countries, and 6.24 in high-income countries. The mode number of grains in each feed ration across all countries was six. Six countries had one grain in their feed ration, which resulted in a Simpson Index of zero. Twenty-one countries had one or two grains in their feed ration.

**Table 4:** Summary, non-inferential, statistics for cereal grain feed rations using reference ration data.

Item	Country income group	Average	Minimum	Maximum	CV
Number of grains in ration	Low	3.04	1	7	0.45
	Lower middle	4.21	1	7	0.40
	Upper middle	5.52	2	7	0.24
	High	6.24	2	7	0.20
Feed ration cost ($ metric ton^−1^)	Low	284	176	781	0.49
	Lower middle	258	177	625	0.41
	Upper middle	216	171	514	0.30
	High	198	171	435	0.21
Simpson’s Index of diversity	Low	0.29	0.00	0.66	0.79
	Lower middle	0.37	0.00	0.70	0.55
	Upper middle	0.34	0.01	0.69	0.67
	High	0.56	0.00	0.76	0.31

*Notes*: Reference ration data are country-specific commodity mixes based on the share of each cereal grain commodity in the cereal grain feed rations [5]. CV is the coefficient of variation, defined as the standard deviation divided by the average. Country income group is from the World Bank Country Group classification. Sample size by country income group: low 27, lower middle 47, upper middle 42, and high 37.

**Fig 1:**
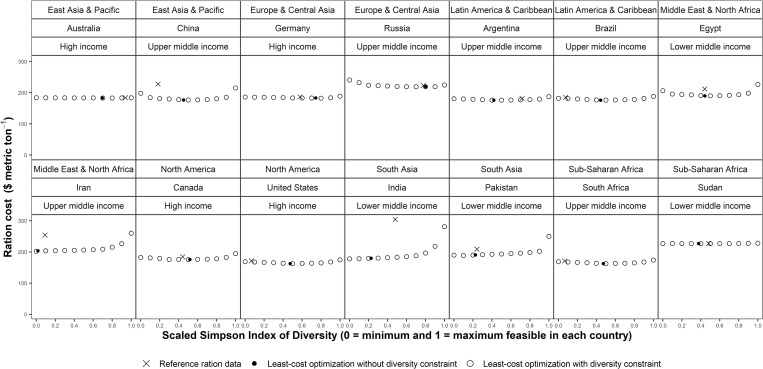
Feed ration cost for different levels of feed ration diversity. Markers labelled ‘Reference ration data’ are country-specific commodity mixes based on the share of each cereal grain commodity in the cereal grain feed rations [5]. Markers labelled ‘Least-cost optimization without diversity constraint’ are simulated rations from the programming model that solves for the least-cost combination of commodities by mass that maintains at least the reference ration nutrient content with no explicit constraint on the level of diversity. Markers labelled ‘Least-cost optimization with diversity constraint’ are simulated rations from the programming model that solves for the least-cost combination of commodities by mass that maintains at least the reference ration nutrient content at specific levels of the Simpson Index of Diversity. Higher values of the Simpson Index indicate a more diverse feed ration. Prices described in Table 3. Scaled Simpson Index is computed using minimum and maximum feasible index value (Table 5). A zero value for the Scaled Simpson Index corresponds to the minimum feasible Simpson Index value in the model for that country. A value of one for the Scaled Simpson Index corresponds to the minimum feasible Simpson Index value in the model for that country.

**Fig 2:**
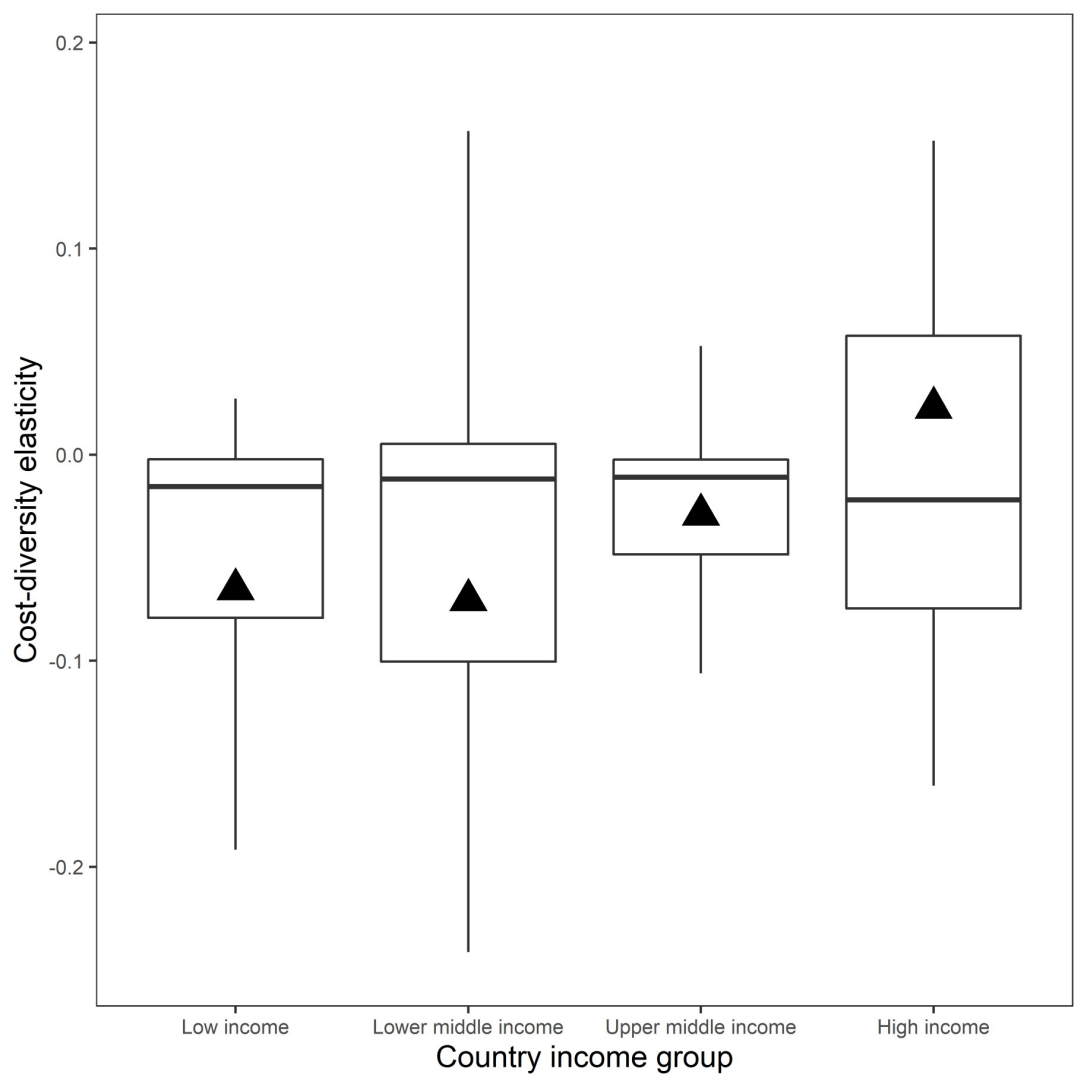
Cost-diversity elasticity around diversity level in the cereal grain reference ration. The cost-diversity elasticity is the percentage change in ration cost in response to a one percent change in the Simpson Index of diversity. Reference rations complied using a global dataset *[5]*. Data reported based on simulations using reference prices in Table *3*. Boxes indicate the interquartile range (IQR). The upper whisker extends from the third quartile upper hinge of the box to the largest value no further than 1.5 × IQR from the upper hinge. The lower whisker extends from the first quartile lower hinge of the box to the smallest value at most 1.5 × IQR from the lower hinge. The line dividing each box shows the median. Triangle markers show the average.

Fig 1 reports the ration costs for different levels of diversity. The black cross markers represent the reference ration data. The simulated least-cost feed rations (hollow circles) often had a lower cost than the cost using the reference ration data. The cost saving differed by country, for example Brazil could reduce ration costs by relatively less than in India (black cross markers versus corresponding value on simulated least cost arc). The hollow circles in Fig 1 are simulated combinations of ration costs and ration diversity, each combination is a least-cost feed ration that has at least the same nutrient content as the reference ration. The solid black circles are the simulated least-cost feed ration when there is no explicit constraint on diversity, i.e., when the programming model is run with the specification in the penultimate column of Table 1. As diversity increased from its minimum level to maximum level costs typically declined and then increased again, although the shape of the cost-diversity relationship differed by country. These relationships resulted in different cost-diversity elasticities (Fig 2). The cost-diversity elasticity associated with a one percent increase in diversity around the reference ration level of diversity ranged from −0.67% to 1.41% (average = −0.02%). The cost-diversity elasticity in low-income countries ranged from −0.24% to 0.17% (average = −0.02%). The cost-diversity elasticity in lower-middle-income countries ranged from −0.67% to 1.05% (average = −0.06%). The cost-diversity elasticity in lower-middle-income countries ranged from −0.38% to 0.20% (average = −0.02%). The cost-diversity elasticity in high-income countries ranged from −0.18% to 1.41% (average = 0.03%). A positive cost-diversity elasticity was present in 32% of countries.

**Fig 3:**
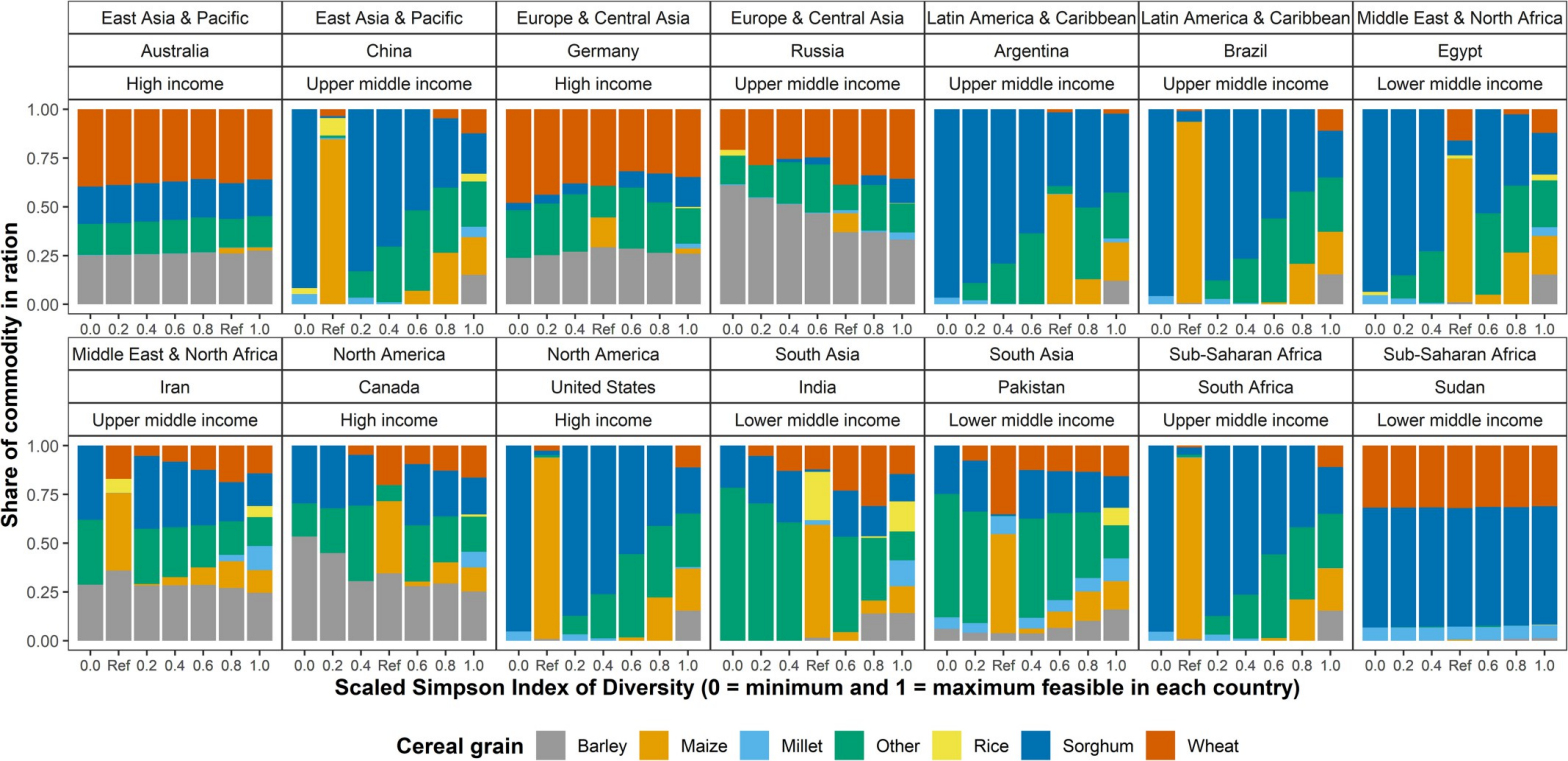
Share of commodity in feed ration by mass for different levels of diversity. Ref on the horizontal axis refers to the reference ration and includes the share of each commodity in cereal grain feed rations reported in the global dataset [5]. Scaled Simpson Index is computed using minimum and maximum feasible index value (Table 5). A zero value for the Scaled Simpson Index corresponds to the minimum feasible Simpson Index value in the model for that country. A value of one for the Scaled Simpson Index corresponds to the minimum feasible Simpson Index value in the model for that country. Data reported based on simulations using reference prices in Table 3.

Fig 3 reports the share of each commodity in the feed rations for the fourteen select countries and Table 5 reports the associated range of Simpson Index values, reference ration costs, and cost-diversity elasticities. Results include the commodity shares for the scenarios listed in Table 1 and the reference ration, and these correspond to a range of diversity levels. The fourteen countries had different levels of diversity in the reference ration as seen by the varying relative position of the reference ration stacked bar for the countries (Fig 3). Maize dominated the reference rations in Brazil, China, South Africa, Brazil, and the United States. India had the most diverse reference ration with more rice in the ration than other countries. As the level of diversity increased, Brazil and the United States reduced the share of sorghum and increased the share of maize in the ration.

**Table 5:** Summary, non-inferential, statistics of feed rations for fourteen select countries.

Country	Simpson Index for reference ration	Minimum feasible Simpson Index	Maximum feasible Simpson Index	Reference ration cost	Cost-diversity elasticity
	(unitless)	(unitless)	(unitless)	($ metric ton^−1^)	(unitless)
Australia	0.73	0.72	0.73	184	1.41
China	0.28	0.16	0.82	227	−0.07
Germany	0.71	0.64	0.75	187	−0.16
Russia	0.69	0.56	0.72	223	0.02
Argentina	0.54	0.07	0.73	182	0.03
Brazil	0.13	0.08	0.78	185	−0.01
Egypt	0.43	0.12	0.82	212	−0.03
Iran	0.68	0.62	0.84	254	0.20
Canada	0.70	0.60	0.82	184	−0.04
United States	0.14	0.09	0.79	172	−0.01
India	0.59	0.34	0.86	304	0.09
Pakistan	0.61	0.53	0.85	209	0.16
South Africa	0.13	0.09	0.78	171	−0.01
Sudan	0.52	0.52	0.53	227	−0.01

*Notes*: Cost-diversity elasticity is calculated using an arc elasticity around the reference ration. Minimum and maximum feasible Simpson Index determined using scenarios summarized in Table 1. Data reported based on simulations using reference prices in Table 3.

## 5. Discussion

Using a global dataset on livestock feed rations and a nonlinear programming model, we identified the cost of changing diversity in cereal grain feed rations for livestock in 153 countries. Our analysis of the dataset highlighted the large inter-country variation in the commodity mixes of these cereal grain feed rations. This reinforces existing literature findings that globally, a wide variety of feed material are offered to livestock, with grains accounting for 13 percent of global feed rations by mass [46]. Although grass and leaves, at 46 percent, dwarfed this 13 percent, our analysis showed that even within the grains category substantial diversity existed in cereal grain feed rations. Our model is based on an annual price and an annual ration cost, i.e., it is not an intertemporal model or intra-annual model and therefore we do not capture intra-annual price variability related to factors such as seasonality.

Some countries had only one or two grains in their feed ration or were reliant on one grain, providing a suggestive indicator of vulnerability to weather and economic shocks. This suggestive indicator of vulnerability reinforces earlier studies that have shown livestock producers often have little flexibility in adjusting their feed mix in response to an increase in the price of a commodity that dominates feed rations, such as maize in the USA [9]. We examined the number of commodities in the reference ration, but there may be other grains available domestically in a country that are currently unused by the livestock sector. The trend we found of an increase in the number of grains in the ration as the income of a country increased follows a range of other trends associated with economic growth, such as the industrialization of agriculture leading to meeting human caloric requirements generating a shift towards more non-food consumption [47] and a shift in human diets away from food staples such as cereal grains [48].

Cross country variation in the commodity mixes of the feed rations we reported is related to a variety of biophysical and socio-economic factors that are largely unrelated to any intent on maintaining diversity in the feed ration. In our current study the nonlinear programming model does not consider these factors; however, if data on context-specific factors were available they could be these included in our model so that the results are more nuanced, at least to the contexts of specific countries. An extensive literature, starting from the 1950s [21, 22], exists on least-cost feed rations that considers the context-specific factors relevant to livestock producers, and includes approaches such as using penalty functions [49, 50]. Our study complements this literature by explicitly examining the cost of diversity within a least-cost framework.

Our results implied that the cost implications of increasing diversity varied substantially by country and that tradeoffs between diversity and costs differed by country. Often an increase in diversity reduced the ration cost, suggesting that marginal changes in diversity around the reference ration may decrease costs in many countries. An increase in ration cost as diversity increases is not ubiquitous, although ration costs typically did increase if diversity increased and approached its maximum feasible value. The shape of the simulated cost curves and the reported cost-diversity elasticities provide the magnitude of the ration cost changes associated with changes in ration diversity. These results highlight where increasing the diversity of feed rations may be financially cheaper, for example, cost-diversity elasticities were, in general, higher in higher income countries than in lower income countries. And in these higher income countries the level of diversity in the reference ration was, in general, already higher than in lower income countries. This result suggests that options to increase diversity are sensitive to initial levels of diversity found in feed rations, which is reflected in the shape of the simulated cost-diversity relationships, and that it is costlier to increase diversity the more diverse the feed ration already is.

Several points for future research related to the economics of livestock feeding are data-intensive. Other feed constraints, beyond total nutrients supplied can exist, for example nitrate poisoning can present a concern for livestock that consume large quantities of annual forage crops (compared with perennial forages). Our nonlinear programming model could accommodate this concern, plus other feed ration constraints for such as for fiber, bulk intake, palatability, or constraints related to pelleting. Additionally, if price and quantity data were available, in a globally-consistent dataset, on other costs that producers might incur if commodity mixes change and on individual feeds within different feed types, we could include these into our programming model.

Our study application focused on grain targeted towards a large commercial manufacturer of livestock feed rations because price data were available. These manufacturers typical have a range of concerns beyond the nutrient content a grain feed ration, such as the availability of feed and food manufacturing byproducts throughout the year, decisions of purchase quantities and timing, and inventory management. Livestock also consume grass, forages, and stover, among others. An open question remains how to quantify the economic value for grass, forages, and stover that uses a globally-consistent method. Assigning a financial cost or opportunity cost to these non-grain feed types would be a useful extension for future research. Also, if we know about preferences for different commodities beyond the cost per unit of nutrient supplied, these could be incorporated into our nonlinear programming model. Given the importance of feed grain production variability and its interaction with prices, dimensions of risk could be included into an extension of the current programming model.

## 6. Conclusion

Researchers have long investigated the benefits of diversity in agricultural production systems and agrifood supply chains; however, in economics there is no free lunch and greater diversity may come at a greater financial or economic cost. Our study presented an approach to calculate the cost of diversity and applied the approach to cereal grain feed rations for livestock in 153 countries. Our main finding was that although in general ration costs increased as ration diversity increased at low levels of diversity (i.e., rations with only one or two commodities) there was potential to increase diversity and reduce costs, as identified by the negative cost-diversity elasticities found, especially in low- and middle-income countries. Tradeoffs between diversity and societal or individual benefits are often deemed ubiquitous. Our study showed that diversity can be increased without a cost increase, this provides insights into what this specific tradeoff may be based on the changes in ration costs as ration diversity changed.
